# Valoración del dolor torácico en pacientes que acuden de forma urgente a atención primaria

**DOI:** 10.1016/j.aprim.2021.102145

**Published:** 2021-07-30

**Authors:** Gloria Albacete Armenteros, Andrés Barrios Recio, Mariano Leal Hernández, José Abellán Alemán

**Affiliations:** a Servicio de Urgencias de Atención Primaria Las Torres de Cotillas. Gerencia, 061, Murcia, España; b Servicio de Otorrinolaringología, Hospital Los Arcos del Mar Menor, Murcia, España; c Centro de Salud San Andrés. Unidad Docente MFyC. IMIB. CESM, Murcia, España; d Centro de Salud de San Andrés, Cátedra de Riesgo Cardiovascular, Universidad Católica de Murcia, Murcia, España

El dolor torácico agudo atraumático es una de las consultas más frecuentes en los servicios de urgencias de atención primaria, y corresponde al 5-15% de la totalidad de estas consultas^1^, ^2^. Resulta de interés analizar los motivos del dolor torácico en los pacientes que acuden a urgencias de atención primaria. El objetivo principal de este trabajo es analizar la incidencia de cardiopatía isquémica en los pacientes con dolor torácico agudo no traumático que acuden a su centro de salud con carácter urgente^1^, ^2^.

Se trata de un estudio observacional descriptivo longitudinal prospectivo cuya muestra es todo sujeto que acude para ser valorado de forma urgente a dos centros de salud de diferentes áreas de la Región de Murcia por presentar dolor torácico agudo atraumático, mayores de 18 años y durante un periodo de tiempo de un mes. Las variables analizadas son edad, sexo, diagnóstico, tipo de dolor torácico (típico/atípico) y derivación a hospital (sí/no). De un total de 2.422 pacientes que acudieron de forma urgente a atención primaria durante ese mes, se identifican 739 personas cuyo motivo de consulta era presentar dolor torácico agudo, que en 200 casos era atraumático. Los resultados obtenidos muestran que el 8% de los pacientes fueron diagnosticados de arritmia: el 81,2% eran mujeres (n = 13) y el 18,8% eran hombres (n = 3). El 30% fueron diagnosticados de ansiedad: el 83,3% eran mujeres (n = 50) y el 16,7% eran hombres (n = 10). El 19% fueron diagnosticados de afección respiratoria: el 76,3% (n = 29) eran mujeres y el 23,7% eran hombres (n = 9). El 11% de los pacientes presentaron dolor torácico de origen incierto: el 54,5% eran mujeres (n = 12) y el 45,5% eran hombres (n = 10). En el 27,5% de los casos el dolor torácico fue de origen osteomuscular: el 54,5% eran mujeres (n = 30) y el 45,5% eran hombres (n = 25). El síndrome coronario agudo se dio en el 4,5% de pacientes, de los cuales el 44,4% eran mujeres (n = 4) y el 55,6% eran hombres (n = 5) (tabla 1).

**Tabla 1:** Distribución de la muestra según sospecha diagnóstica y en relación con el sexo

Diagnóstico	Total	Hombre n (%)	Mujer n (%)	DTT n (%)	DER n (%)
Arritmia	16 (8%)	3 (18,8%)	13 (81,2%)	12 (75%)	13 (81,3%)
Ansiedad	60 (30%)	10 (16,7%)	50 (83,3%)	10 (16,7%)	0 (0%)
Afecciones respiratorias	38 (19%)	9 (23,7%)	29 (76,3%)	0 (0%)	0 (0%)
Dolor torácico incierto	22 (11%)	10 (45,5%)	12 (54,5%)	10 (45,5%)	22 (100%)
Osteomuscular	55 (27,5%)	25 (45,5%)	30 (54,5%)	0 (0%)	0 (0%)
Síndrome coronario agudo	9 (4,5)	5 (55,6%)	4 (44,4%)	7 (77,8%)	9 (100%)

% DER: porcentaje de pacientes derivados a urgencias hospitalarias.

% DTT: porcentaje de pacientes con dolor torácico típico.

La relación entre las variables no fue estadísticamente significativa (p > 0,152). Cuando se analiza a los pacientes respecto a si el dolor torácico es de características típicas o no, resulta que de los pacientes diagnosticados de arritmias (8%), el 75% presentaban dolor torácico típico (n = 12); de los diagnosticados de ansiedad (30%), el 16,7% presentaban dolor torácico típico (n = 10). De los diagnosticados de afección respiratoria (19%), ninguno de ellos (0%) presentaba dolor torácico típico; de los diagnosticados de dolor torácico de origen incierto (11%), el 45,5% presentaban dolor torácico típico (n = 10). Cuando el diagnóstico era dolor torácico de origen osteomuscular (27,5%), ninguno de ellos (0%) presentaba características típicas. En los diagnosticados de síndrome coronario agudo (4,5%), el 77,8% presentaban dolor torácico típico (n = 7), siendo la relación entre las variables estadísticamente significativa (p < 0,05).

Respecto a su derivación a urgencias hospitalarias, el 81,3% de los pacientes diagnosticados de arritmia (13 pacientes) fueron derivados al hospital. En el caso de los pacientes con ansiedad, afección respiratoria o dolor de características osteomusculares, ninguno precisó derivación. La totalidad (100%) de los pacientes con dolor torácico de origen incierto o síndrome coronario agudo fueron derivados a urgencias hospitalarias, siendo la relación entre las variables estadísticamente significativa (p < 0,05). Se compararon los resultados obtenidos con los de otros estudios realizados previamente, aunque estos son escasos, ya que la mayor parte de la literatura encontrada se ha desarrollado en medio hospitalario y los estudios van enfocados a la valoración del dolor torácico, su diagnóstico y el posterior tratamiento, haciendo menos hincapié en la prevalencia, como sí se ha hecho en este estudio^3^, ^4^, ^5^, ^6^. En conclusión, se puede afirmar que los síndromes coronarios agudos diagnosticados y valorados en el servicio de urgencias de atención primaria eran escasos, y menos de la cuarta parte de los pacientes valorados por dolor torácico agudo son derivados a los servicios de urgencias hospitalarios.

## Conflicto de intereses

Los autores declaramos que no hay ningún tipo de conflicto de intereses ni responsabilidades éticas.
